# First Report of the Pepper Fruit Fly *Atherigona orientalis* (Schiner 1968) (Diptera: Muscidae) Infesting Commercial Pepper Crops in Greece

**DOI:** 10.3390/insects14040393

**Published:** 2023-04-18

**Authors:** Emmanouil Roditakis, Katerina Kremi, Kyriaki Mylona, Vasilios Georgousis, Dimitrios N. Avtzis, Konstantinos B. Simoglou

**Affiliations:** 1 Department of Agriculture, School of Agricultural Sciences, Hellenic Mediterranean University, Estavromenos, 71410 Heraklion, Greece; 2 Institute of Agri-Food and Life Sciences, Hellenic Mediterranean University Research Centre, 71410 Heraklion, Greece; 3 Biotopio P.C., 72200 Ierapetra, Greece; 4 Forest Research Institute, Hellenic Agricultural Organization Demeter, Vassilika, 57006 Thessaloniki, Greece; 5 Department of Quality and Phytosanitary Inspections, Rural Economy and Veterinary Directorate, 66133 Drama, Greece

**Keywords:** fruit fly, pepper, diptera, *Atherigona orientalis*, Greece, damage

## Abstract

**Simple Summary:**

The pepper fruit fly *Atherigona orientalis* is a cosmopolitan tropical dipteran pest. The biology of *A. orientalis* is primarily associated with rotting fruits and vegetables, but also with vertebrate and invertebrate carrion and manure. In recent years, *A. orientalis* has also been reported as a major pest of pepper fruits. Hereby, we report, for the first time in Greece and, to the best of our knowledge, in Europe, cases of pepper fruit fly damaging pepper fruits in commercial greenhouse crops (in Crete in 2022). The possible implications and concerns associated with the occurrence of this novel pest are discussed.

**Abstract:**

The pepper fruit fly *Atherigona orientalis* (Schiner 1968) (Diptera: Muscidae) is a cosmopolitan tropical pest which has been recently recorded in several European countries. The biology of the pest has been primarily associated not only with decomposing fruits and vegetables, but even vertebrate and invertebrate carrion, dung and faeces. Relatively recently, *A. orientalis* has been reported as a primary pest of pepper fruits as well. In this short communication, we report, for the first time in Greece and, to the best of our knowledge, in Europe, cases of pepper fruit fly damage to pepper fruits in commercial greenhouse crops (in Crete in 2022). In this direction, possible implications and concerns regarding the occurrence of this pest in Crete are discussed.

## 1. Introduction

Pepper (*Capsicum annuum* L.) is one of the most popular vegetables in the Greek diet, with the majority of Greek residents frequently consuming peppers [1]. The vast majority of greenhouse crops in Greece are vegetables, while the most important greenhouse vegetable crop species are tomatoes and cucumbers, followed by peppers [2,3]. *Atherigona orientalis* is common in tropical and subtropical areas of the Old and New World. In particular, the pepper fruit fly can be found from the USA southwards to Argentina and Chile; in the Canary Islands, in North Africa and the entire Afrotropical Region; the Middle East and Turkey; and in China and Japan through to Australia and the Pacific [4,5,6,7]. In Europe, it has been reported in Malta, Cyprus [8] and, most recently, in Spain in forensic entomological baits; these are considered to be the first occurrences in the European mainland [9].

The genus *Atherigona* Rondani includes two subgenera: (i) *Atherigona* Rondani, the larvae of which are phytophagous and feed primarily on wild and cultivated Poaceae species; and (ii) *Acritochaeta* Grimshaw, which are mostly saprophagous species and live in a wide range of decaying plant and animal material [4,5]. Despite belonging to the subgenus *Acritochaeta*, the pepper fruit fly (*A. orientalis*) can sometimes be a primary pest of specific agricultural crops [10,11] and is a widespread pest of peppers, tomato and sorghum in various African countries and in southern Asia [7]. It is a highly polyphagous species, as the larvae develop not only in live and decaying plant materials, faeces and carrion, but it also predates on the living larvae of other insect species such as *Bactrocera* spp. and *Dacus* spp. [12]. Several plant species have been reported as major hosts of *A. orientalis*, namely cabbage and cauliflower (*Brassica oleracea* L.), pepper (*Capsicum annuum* L.), orange (*Citrus sinensis* (L.) Osbeck), melon (*Cucumis melo* L.), tomato (*Solanum lycopersicum* L.) and beans (*Phaseolus* spp.) [7,8,12].

In peppers, in particular, *A. orientalis* lays eggs either individually or in clusters on or under the calyx of the fruits [11,13]. The eggs are white and about 1.0 mm long, and are laid in cracks on the surface of fruits (ripe or even rotten), near the egg masses of other insects, or even in carrion and faeces, since the females lack a sharp, strong ovipositor that could facilitate the piercing of harder tissues [10]. During high infestation levels, even cuticle cracks, blossom ends and receptacles are utilized for egg deposition [13,14]. The newly hatched first instar enters the mesocarp, causing substantial damage to the internal tissues [15,16], leading to the shedding of the pepper fruit [14]. A full description of the immature stages was given by Couri and Araújo, Hibbard and Overholt, and Grzywacz and Pape [10,17,18] and of the adults by Pont [4], and Suh and Kwon [7,12]. Ogbalu [19] suggested that particular traits of pepper fruit varieties influenced the attractiveness and infestation by the pepper fruit fly, such as the small size and red colour of fruits, the lack of grooves, the tight calyx and thin epicarp, the non-fleshy mesocarp and, finally, the high pungency and texture. Young, unripe and mature pepper fruits are all damaged under heavy infestations [19].

In the communication, we report damage by the pepper fruit fly *A. orientalis* in commercial greenhouse crops for the first time in Greece (in Crete in 2022) and, to the best of our knowledge, in Europe as well. This finding is of particular importance for the invasion pattern of the pest, as the pepper fruit fly has been recently reported in Europe and South-Western Asia [9,20].

## 2. Materials and Methods

### 2.1. Sampling Locations

In April 2022, complaints from greenhouse vegetable growers were reported locally about extensive damage by unknown insect pests in an organic sweet pepper greenhouse (*Capsicum annuum* hb. Palermo RZ F1 and Idolisa RZ F1, 0.20 Ha) in the Tympaki rural area (Central Southern Crete, Greece). Approximately at the same time (May 2022), a second infestation of pepper fruits (cv. Florinis) by unknown pests, with a lower damage impact, was reported in a greenhouse (0.65 ha) in the area of Stomio (Psalidena) Ierapetra (Southeastern Crete). Both sampling sites, Tympaki (35°05′24.5″ N, 24°46′36.1″ E) and Stomio (35°00′58.6″ N, 25°40′10.0″ E), are coastal areas less than 70 m in altitude, characterized by a Mediterranean climate. Specimens of infested pepper fruits from both locations (approximately 20–25 symptomatic fruits) were transferred to the Laboratory of Entomology (Hellenic Mediterranean University, Heraklion Crete, Greece) for further examination. The collected infested peppers from both locations consisted mostly of ripe fruits prior to or at the harvesting stage.

### 2.2. Insect Detection and Rearing

After a thorough examination of the infested pepper fruits under a high magnification stereoscope (Olympus SZ6), several larvae of dipterous pests were detected feeding in the inner part of the pepper fruit. Infested pepper fruits were placed in open-top boxes with multiple layers of tissue paper covering the inner part of the box, in order to absorb any fluids of fruit decomposing. The boxes were maintained in large, ventilated insect-proof cages under controlled conditions (25 ± 2 °C, 60–65% relative humidity, 16 h L:8 h D). Larvae utilized the layers of the tissue paper, where they pupated. The larvae were monitored daily with minimal interventions (i.e., removal of deteriorating tissues) supporting insect development. Approximately 8–10 days later, adult flies started to emerge.

### 2.3. Species Identification

Species identification was based both on morphological and molecular markers. Species-specific taxonomic identification keys were used [4,12] for classical morphological identification. Molecular identification utilized a DNA barcoding protocol of the respective mtDNA locus. For this purpose, DNA was extracted the whole body of two individuals separately using a PureLink^®^ Genomic DNA kit (Invitrogen, Waltham, MA, USA) and following the manufacturers’ instructions. A polymerase chain reaction (PCR) was then run in 25 μL volumes with the primers LCO (5′-GGTCAACAAATCATAAAGATATTGG-3′) and HCO (5′-TAAACTTCAGGGTGACCAAAAAAT-3′), which amplify a 658 bp fragment of the cytochrome oxidase subunit I (COI) gene of mtDNA [21]; the conditions were described by Avtzis et al. [22]. Purification of the PCR products was carried out with a PureLink^®^ PCR Purification Kit (Invitrogen) according to the manufacturer’s protocol, and then the purified products were sequenced in the automated sequencer ABI3730XL of CeMIA Company (Larisa, Greece), using the same primers as the ones used for the PCR. Finally, the sequences were initially visualized with Chromas Lite software version 2.01 and then blasted against the NCBI GenBank database.

### 2.4. Estimation of the Damage Level

The damage level of the pepper fruits was estimated in the greenhouse from the Stomio area. Approximately 2000 fruits were examined macroscopically for typical symptoms of deterioration. The damaged fruits were separated and the percentage of damage was estimated. In the Tympaki area, the damage was a result of mixed species infestation. It was not possible to discriminate the damage levels per species; therefore, this particular parameter was not evaluated in that location.

## 3. Results

### 3.1. Species Identification

The majority of adults (42 of 44) emerging from sample of the Tympaki rural area were morphological identified as the Mediterranean fruit fly *Ceratitis capitata* (Wiedemann 1824) (Diptera: Tephritidae), which is already known to infest pepper fruits [23]. However, two adults differed significantly at a morphological level, bearing no resemblance to any species detected before in the region, suggesting a mixed species infestation.

**Figure 1:**
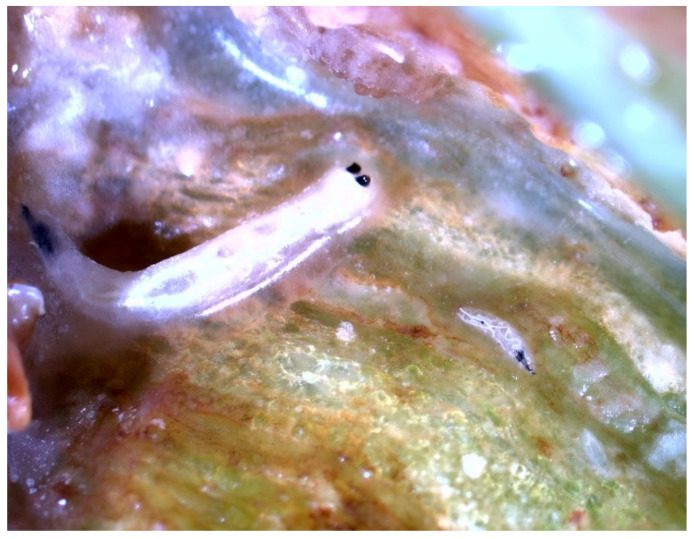
*Atherigona orientalis* specimen: second and third instar larvae on pepper fruit.

**Figure 2:**
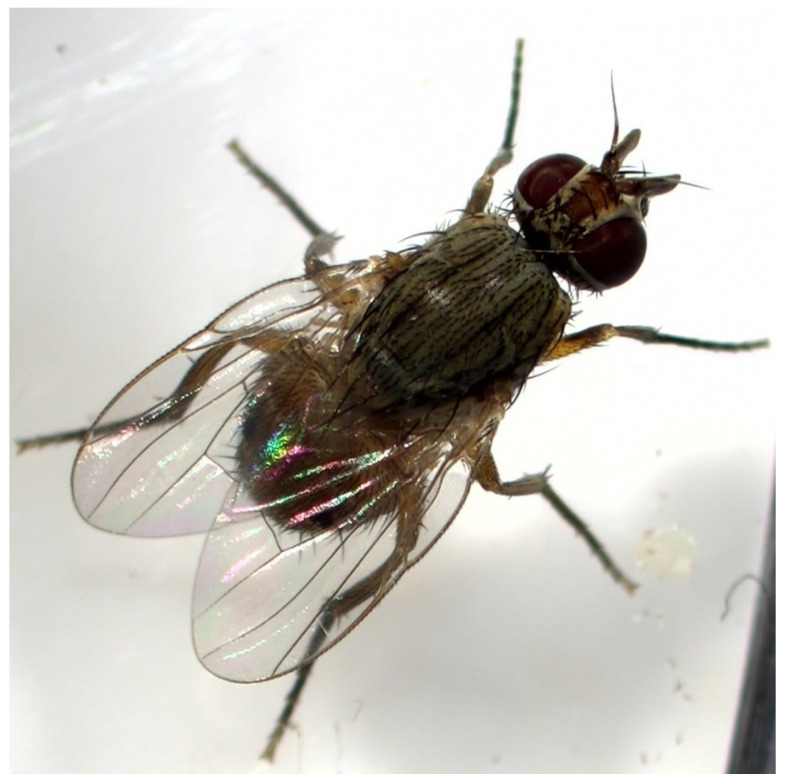
Adult *Atherigona orientalis*.

In the Stomio sample, the larvae resembled those of the unknown species found at the Tympaki area earlier in the cropping season (April 2022). The larvae were exclusively feeding in the inner parts of the pepper fruits. Approximately six to seven larvae from three different instars per fruit were detected. All instars had a distinct cephaloskeleton and a pair of distinctive, black posterior spiracles (Figure 1), indicating a potential infestation by *Atherigona* species. Eggs were found on the calyx and on the smooth surface near the calyx. Up on adult emergence, using taxonomic identification keys [4,12], all adults from the Stomio sample were identified as the pepper fruit fly (Figure 2).

Furthermore, DNA barcoding of the samples from both infested locations confirmed the aforementioned finding. Blasting against the NCBI GenBank database revealed 99.42% resemblance of our sequences with EU627707, which corresponds to *A. orientalis*.

### 3.2. Description of the Damage

The feeding activity of the larvae on the pepper fruits resulted in tissue deterioration, followed by an external colour change, tissue softening, detachment of the external epidermis and loss of vigour. Secondary microbial infestations have been observed in some cases. Only fruits that suffered extensive internal tissue degradation exhibited typical symptoms externally. Early-stage infestation was not possible to detect via visual examination.

### 3.3. Estimation of the Damage Level

In the Tympaki area, it was not possible to evaluate the damage levels by *A. orientalis*, since a mixed species infestation occurred. The only information available is that damages were first detected on hb. Palermo and expanded on hb. Idoliza, leading to extensive crop damage overall (>50%).

Damages in the Stomio greenhouse was mostly abundant in ripe (red coloured) pepper fruits. According to the number of fruits exhibiting symptoms, the damage level was estimated at 2%, although the actual damage should be considered to be higher, since early-stage infestations do not exhibit external symptoms. However, it was not possible to examine asymptomatic fruits via dissection for early-stage infestations, since the asymptomatic fruits consisted of the commercial product of the greenhouse.

## 4. Discussion

This is the first report of *A. orientalis* infesting and damaging greenhouse peppers in Europe. In the pepper crop from the Tympaki area, *A. orientalis* larvae were found together with the larvae of the Mediterranean fruit fly in the interior of the pepper fruits. The pepper fruit fly often lays its eggs on the oviposition sites of other insects and even scavenges Tephritidae larvae, so this behaviour is well known [5,10]. However, in the case of the infestation detected in a greenhouse pepper crop in Stomio (Psalidena), Ierapetra rural area, the presence of larvae of other insect species was not observed and, therefore, the damage was attributed purely to *A. orientalis*. The occurrence of *A. orientalis* in Crete, the main greenhouse area of Greece, has significant implications for the pest management schemes implemented in pepper crops. Until recently, the major pests of peppers, such as whiteflies, aphids, thrips and leafminers, were controlled by employing IPM approaches with minimum/no use of insecticides (Roditakis, unpublished data). However, none of the approaches implemented for the aforementioned pests are known to be effective in controlling Diptera species such as *A. orientalis*. Ogbalu et al. [13] suggested that *A. orientalis* females prefer pepper cultivars where the fruits have a raised/open calyx and grooves over those lacking these characteristics. Overall, the surface morphology and characteristics of pepper fruits influence their choice as oviposition sites; therefore, different varieties can be studied as a cultural control method of the pest. *Atherigona orientalis* is also known to infest tomato plants (*S. lycopersicum* L) which is a significant greenhouse and field crop cultivated in both areas. It is therefore vital to disseminate information to agronomists and greenhouse growers around the Mediterranean basin in order to facilitate an early warning system that will safeguard both the quality and quantity of the crops.

Cabrera-Cánoves et al. [9] suggested that the establishment of *A. orientalis* in mainland Europe is probably based both on the current climatic conditions and on the indication of potential climate warming in the region [24]. Skendžić et al. [25] thoroughly reviewed the potential links between climate change and crop pest insects. They reported that the response of insect pests to an increased temperature and changeable precipitation patterns, the expansion of the insects’ distribution, their increased overwintering survival and the increased risk of invasive alien insect species reduced the effectiveness of biological control agents; these are examples of the reported possible impacts of climate change on insect pests. Since this is a multidimensional issue [26], it is not possible to draw conclusions and, therefore, additional research is needed to confirm any relationship between the occurrence of *A. orientalis* on the European continent and climate change.

Overall, the establishment and the potential future expansion of *A. orientalis* in the Mediterranean/European region, the main production area of vegetables in the EU, is of particular concern for future pest control schemes. Currently established IPM schemes for greenhouse peppers do not include actions for the control of Diptera. Sprays with conventional insecticides that exhibit high efficacy against Diptera (such as diamides or spinosyns) could serve as commercially available tools for the control of *A. orientalis* [27]. However, such approaches may interfere with the performance of beneficial insects currently used under the IPM schemes for peppers. Potentially, the development of efficient monitoring tools in combination with IPM-compatible lure and kill techniques may be a more sustainable approach. At this stage, the awareness of the stakeholders of the potential implications for the sustainable production of pepper fruits of the novel pest is of key importance, while, in parallel, collaborations between industry and academia should be established for the development of IPM-compatible control tools.

## Data Availability

All data are included in the manuscript.
